# Individually Tailored Internet-Based Cognitive-Behavioral Therapy for Daily Functioning in Patients with Parkinson’s Disease: A Randomized Controlled Trial

**DOI:** 10.3233/JPD-191894

**Published:** 2020-04-03

**Authors:** Martin Kraepelien, Robert Schibbye, Kristoffer Månsson, Christopher Sundström, Sara Riggare, Gerhard Andersson, Nils Lindefors, Per Svenningsson, Viktor Kaldo

**Affiliations:** a Department of Clinical Neuroscience, Centre for Psychiatry Research, Karolinska Institutet & Stockholm Health Care Services, Region Stockholm, Stockholm, Sweden; b Department of Clinical Neuroscience, Division of Psychology, Karolinska Institutet, Stockholm, Sweden; c Department of Dental Medicine, Division of Orthodontics and Pediatric Dentistry, Karolinska Institutet, Stockholm, Sweden; d Center for Lifespan Psychology, Max Planck Institute for Human Development, Berlin, Germany; e Max Planck UCL Centre for Computational Psychiatry and Ageing Research, Berlin, Germany/London, United Kingdom; f Department of Psychology, University of Regina, Regina, Canada; g Department of Learning, Informatics, Management and Ethics, Karolinska Institutet, Stockholm, Sweden; h Department of Behavioural Sciences and Learning, Linköping University, Linköping, Sweden; i Department of Clinical Neuroscience, Section of Neurology, Karolinska Institutet, Stockholm, Sweden; j Department of Psychology, Faculty of Health and Life Sciences, Linnaeus University, Växjö, Sweden

**Keywords:** Internet, psychological treatment, cognitive-behavioral therapy, Parkinson’s disease

## Abstract

**Background:**

Parkinson’s disease (PD) is often associated with psychological distress and lowered daily functioning. The availability of psychological interventions tailored for people with Parkinson is very limited.

**Objective:**

To study if guided individually-tailored internet-based cognitive behavioral therapy (ICBT) provide additional value to standard medical treatment for PD.

**Methods:**

Seventy-seven individuals with PD and self-reported problems with general function measured with the Work and Social Adjustment Scale (WSAS > 15) were randomized to 10 weeks of either ICBT combined with standard medical treatment, or standard medical treatment plus being on waitlist to ICBT (CONTROL). Change in the main outcome WSAS, as well as secondary measures such as quality of life, depression, anxiety and insomnia symptoms were investigated post treatment.

**Results:**

Participants receiving ICBT reported significantly higher functioning after treatment (WSAS group difference –4.56, controlled effect size *g* = 0.69, significant group by time interaction, W*χ*^2^= 26.23, *p* = 0.001). However, only around one third of participants in the treatment group were classified as treatment responders, defined as having a 30% reduction on the WSAS post treatment. Patient involvement and ratings of ICBT credibility were high. Symptoms of anxiety, depression and insomnia symptoms were significantly lower after treatment compared to CONTROL. There were also positive effects on Parkinson-specific function and quality of life in the treatment group.

**Conclusions:**

ICBT as an addition to standard medical treatment was credible and improved functioning for some individuals with PD. Still, the treatment needs further development in order to help a larger proportion of individuals with PD.

**Trial registration number:**

ClinicalTrials.gov NCT02627885.

## INTRODUCTION

Although Parkinson’s disease (PD) is often primarily associated with motor symptoms, there are also common psychological and other non-motor symptoms that strongly affect daily functioning and quality of life. A majority of people with Parkinson (PwP) have symptoms of depression and/or anxiety [1], and many also experience sleep disorders like insomnia [2, 3]. There is suggestive evidence that psychological treatments based on cognitive-behavioral approaches can alleviate depressive and anxiety symptoms in PD [1, 4], reduce impulse control behaviors [5], and reduce caregiver burden [6]. Self-help interventions like telephone- or internet-based treatments have the potential to extend the reach of these treatments. Evidence for self-help interventions adapted to PwP is, however, scarce. Recently, there have been promising smaller non-randomized studies of telephone and video-delivered cognitive-behavioral interventions for depression associated with PD [7–9], but larger randomized trials of remotely delivered self-help are lacking.

Internet-based cognitive behavioral therapy (ICBT) is a guided self-help treatment format that has been shown to be as effective as face-to-face cognitive behavioral therapy for many symptoms, like depressive and anxiety symptoms, in the general adult population [10]. For these conditions, ICBT is regarded as a cost-effective type of intervention due to the limited therapist-time required [11]. Although there are ICBT programs adapted for people with chronic conditions in general [12], there is currently no ICBT program adapted for PD.

In an earlier uncontrolled feasibility study by our research group [13], an individually tailored ICBT-program for depression and anxiety in PD showed preliminary encouraging effects on depressive symptoms. At the same time, treatment adherence and satisfaction were somewhat low compared to a similar program for depression in the general adult population [14]. Some comments from participants in the feasibility study suggested a need for the treatment content to be more adapted for PwP, and for a more user-friendly interface of the treatment platform [13]. Hence, a new version of the ICBT-program was developed focusing more on PwP-related daily functioning. Examples of daily functioning in this context is the ability to be physically active, the ability to manage your home, to do leisure activities, and to manage healthy social relationships. The new, supposedly more user-friendly, interface was based on an internet platform aimed to support face-to-face treatments [15, 16].

The aim of the present study was to, evaluate the effects of an ICBT-program for PwP on daily functioning, in comparison to being on a waitlist to ICBT, as well as to further explore involvement, treatment satisfaction and patients’ subjective evaluations. The aim was also to investigate the effects on secondary outcome measures such as depressive symptoms, anxiety, insomnia symptoms, and quality of life.

## METHODS

PwP reporting problems with general function were randomized (1 : 1) to receive either 10 weeks of ICBT as an adjunct to their standard medical treatment, or to standard medical treatment plus being on waitlist to ICBT (CONTROL). The random allocation was done by an independent research nurse using sequentially numbered, sealed envelopes. Outcomes were assessed with questionnaires at pre-, mid- (5 weeks), and post-treatment (10 weeks).

The study was approved by the Regional Ethics Review Board in Stockholm, Sweden (2015/1938-31/4) and registered at ClinicalTrials.gov (NCT02627885).

### Participants

**Fig.1:**
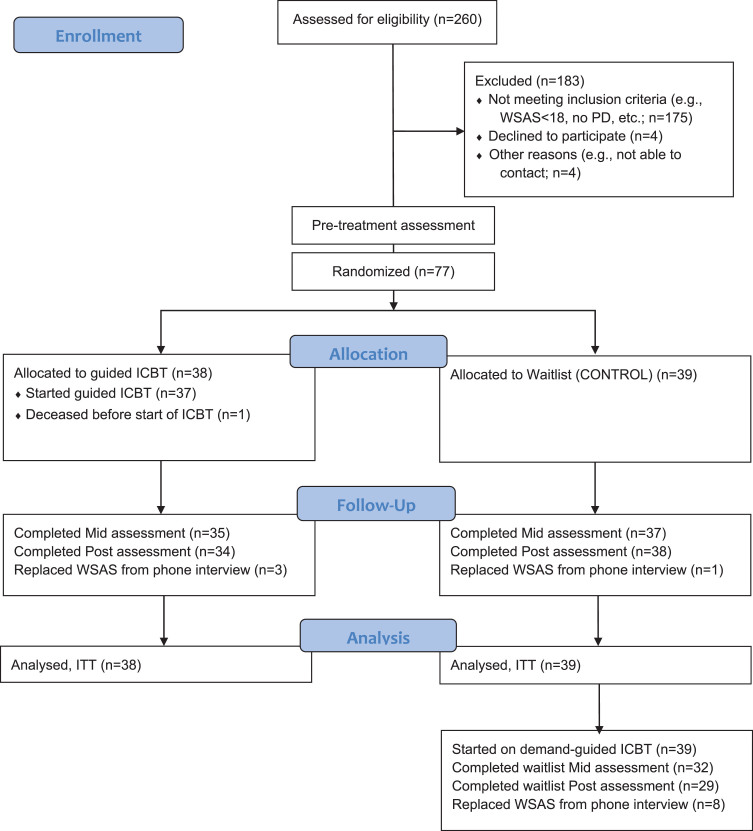
CONSORT flow diagram of the participants’ progress through the study of ICBT for general function in PD. CONSORT, Consolidated Standards of Reporting Trials; ICBT, internet-based cognitive–behavioural therapy; PD, Parkinson’s disease; WSAS, Work and Social Adjustment Scale; ITT, Intention To Treat.

Participants were recruited between February and April 2016 with the help of ads and the official newsletter of the Swedish Parkinson society, through a secure webpage with internet-based screening questionnaires. Two hundred and sixty potential participants completed the screening assessment (Fig. 1). The inclusion criteria included (1) diagnosed PD; (2) significant amount of self-reported problems with general function defined as 18 points or more on the Work and Social Adjustment Scale (WSAS) [17]; (3) regular access to at least one internet-enabled computer, tablet or smartphone, and being able to receive text messages (SMS). The exclusion criteria included (1) substance or alcohol abuse; (2) psychotic disorder, bipolar disorder or other serious psychiatric disorder that could prevent taking part of the intervention; (3) practical obstacles that hinders participating in the intervention, such as not having enough time, or having too severe PD symptoms, to be able to actively participate in the study; (4) high suicide risk, self-rated or based on a standardized clinical interview. After the internet-based screening the participants who fulfilled the inclusion criteria were contacted to be interviewed by a psychologist in clinical supervised training or a clinical psychologist. Conducted over telephone, the aim of the interview was to again screen for the inclusion and exclusion criteria and decide in accord with the participant on a preliminary plan for the personalized treatment modules if the participant were to be included. The interview included the MINI semi-structured interview [18] to assess current and past psychiatric disorders. Final inclusion in the study was then decided after discussion with the interviewer and the study coordinator and clinical supervisor (MK). If included, there were no restrictions to other simultaneous pharmacological or psychological treatments since the ICBT was being evaluated as an adjunct treatment.

Based on the preliminary effects in the feasibility study, we aimed to include at least sixty-four participants with an expected between group effect size of 0.7, *α*= 0.05 and 80% power. Because of very high interest in the trial after the Parkinson society’s newsletter, and the high capacity of letting several participants go through ICBT at once, thirteen extra participants were included before the recruitment to the study stopped. A total of seventy-seven participants were finally included in the study and were randomized after having completed the internet-based pre-treatment assessment and a telephone assessment.

### Primary outcome measure

Primary outcome measure was the self-rated five-item WSAS, measuring impairment in functioning in five domains: work, home management, social leisure activities, private leisure activities and relationships with others [17]. WSAS is widely used as an outcome measure in interventions for mental health, since it allows for a flexible specification of which condition the individuals rate their functioning in relation to, e.g., “*Because of my Parkinson*’*s,* my ability to work is  ...  ”, and since it is sensitive to treatment-related change [17]. WSAS has been used as an outcome in an earlier trial with PwP [5] but has not been specifically validated with a PwP-sample. The WSAS was administered by phone in cases where questionnaire data was missing [19].

### Secondary outcome measures

The secondary outcome measures included the Hospital Anxiety and Depression Scale [20] with separate subscales measuring anxiety (HADS-A) and depression (HADS-D), the Insomnia Severity Index (ISI) [21] measuring insomnia severity, the Parkinson’s Disease Questionnaire-8 (PDQ-8) [22] measuring functioning and well-being specifically for PwP, the World Health Organization Disability Assessment Schedule 2 - 12-item (WHODAS-2) [23] measuring general disability, the Brunnsviken Brief Quality of life scale (BBQ) [24] measuring quality of life, the Stanford Self-Efficacy for Managing Chronic Disease (SSES6) [25] measuring self-efficacy in relation to a chronic disease. Clinical Global Impression - Severity and Improvement scales (CGI-S, CGI-I) [26] were assessed by phone, post treatment, in the treatment group only, by a clinical psychologist or a clinical psychology student (Master’s level) under supervision, who used the participant’s communication and questionnaires to evaluate severity and improvement on a scale from 1 to 7 according to the official CGI instructions. Number of adverse events and use of other treatments during the treatment period were self-reported post treatment, together with subjective qualitative reports of adverse, and positive, events during treatment. Measures of physical activity and valued activities were not used due to problems in the digital questionnaires.

### Feasibility measures

Patient involvement in the intervention was defined as the number of completed modules as well as numbers of sent and received messages by the participant. Since the treatment was 10 weeks long and a treatment module was used during one treatment week, the maximum number of modules can be considered as 10. The first four modules were considered to be the essential parts of treatment and a completion of these was used as a proxy for good enough engagement. Treatment satisfaction was measured with the Client Satisfaction Questionnaire-8 item version (CSQ-8) which ranges from 8 to 32 points. [27] A score of 8–13 was considered poor, 14–19 was considered fair, 20–25 was considered good, and 26–32 was considered excellent. [28] Subjective evaluation of the treatment by written free text comments constituted a qualitative evaluation of the treatment. The benefit of using the activity meter to increase physical activity was self-rated post treatment on a scale from 0–4 where 0 stood for no benefit and 4 for very much benefit.

### Intervention (ICBT)

All patients in ICBT also continued to follow the plan for standard medical treatment they had before entering the study. Similar to the program used in our feasibility study [13], the program was individually tailored and consisted of both mandatory and optional treatment modules (Table 1). Inspired by the feasibility study [13] and another individually tailored ICBT intervention targeting depression and anxiety [29] the tailoring-procedure resulted in an individual treatment plan consisting of 5 mandatory, and 1–5 optional treatment modules. During the pre-treatment interview, the participants made an informed choice on which optional modules to include.

**Table 1:** Intervention content

Treatment module	Mandatory/optional	Homework assignment
Module 1: Introduction	Mandatory	On time-diary, identifying values
Module 2: Physical and valued activity	Mandatory	Activity scheduling, use activity meter
Module 3: Stress, anxiety and avoidance	Mandatory	Confront an avoidance behavior
Module 4: Problem solving strategies	Mandatory	Problem solving, acceptance
Assertive communication	Optional	Practice assertive communication
Cognitive distortions	Optional	Cognitive reappraisal
Existential questions	Optional	Imagine 100th birthday party
Mindfulness training	Optional	Mindfulness exercises with audio
Pain	Optional	Pain acceptance
Panic and agoraphobia I	Optional	Hyperventilation test, *in vivo*-exposure
Panic and agoraphobia II	Optional	Controlled breathing, interoceptive exposure
Relaxation training	Optional	Progressive relaxation training with audio
Rumination	Optional	Identify rumination, activity scheduling
Sexuality and intimacy	Optional	Reflect on sexuality with partner or self
Sleep and circadian rhythm	Optional	Sleep restriction and stimulus control
Social anxiety	Optional	*In vivo*-exposure
Worry	Optional	Worry time
Last module: living actively	Mandatory	Summary, plan for the future

The modules were accessed by the participant one at a time, in a planned rate of one module per week. A module consisted of educative texts, interactive forms and a homework exercise and were 2438 (SD = 836) words long on average, compared to around 3000 words in the feasibility study.

Parts of the newly developed ICBT-program was inspired by representatives from the PwP community, especially an expert patient (SR), and previous participants’ comments. For example, this led to the development of a new type of *on/off*-diary for the registration of fluctuating Parkinson symptoms [30]. where the PwP would use the diary to register *on*-time (periods when the medication is working well) only, and not focus on *off*-periods (periods when the medication is not working well).

Other additions included a new focus on increasing valued activities of daily living and increasing physical activity, rather than on decreasing depression and anxiety, optional modules on existential questions and sexuality, and a digital activity meter (pedometer) and instructions on how to use it to promote physical activity. The activity meter used a visual cue to prompt low-intensity physical activity (corresponding to a few minutes of walking) when physically inactive for an hour or more.

The weekly therapist guidance consisted of written messages within the secure treatment platform. Guidance focused on encouraging the patient to engage with key treatment components. The aim was that the therapist spent 15 minutes per patient per week. [31] Therapists were instructed to reply to completed homework assignments and to participants’ questions and to remind patients having been inactive for a week. If the patient had technical problems or was not active online, reminders and technical assistance were given through phone calls and SMS.

### Control group (CONTROL)

Individuals in CONTROL received information about being on the waitlist for ICBT and an approximate date on when to start. During this period, they followed their plan for standard medical treatment. The delayed treatment participants in CONTROL later received was similar to the program in the intervention group except for the guidance being on demand only, and the text in the treatment modules was adjusted to reflect this.

### Statistical analyses

The statistical analyses were conducted in SPSS version 25 (IBM Corp) and STATA version 15 (StataCorp). Intention-to-treat analyses were performed. Five participants (6%) had missing data post treatment. Four of these missing values for the main outcome self-rated WSAS could be replaced by WSAS conducted by telephone, bringing the amount of missing post measurements to one for WSAS (Fig. 1). Generalized Estimating Equations (GEE) with exchangeable correlation structure were used to create estimated marginal means based on all available time points for each measure, and to test for group differences in the rate of symptom reduction (group×time interaction). Both between- and within-group effect sizes were calculated as Hedge’s *g* and presented with 95% confidence intervals (CI). As an additional analysis, a Bayes factor (B) was calculated to assess the strength of evidence on the main outcome WSAS between groups at post treatment [32]. The calculated Bayes factor used no difference between the groups at post-treatment as the null hypothesis (H0) and an alternative hypothesis (H1) of a difference in favor of ICBT compared to CONTROL of 3.3 points, representing a moderate effect size. A B of >3 supports H1, *B* < 1/3 supports H0 and a B between 1/3 and 3 represents insensitive data [32].

A dichotomous variable was created for individuals with at least a 30% reduction on the WSAS, representing a treatment responder. Thirty percent reduction was chosen rather arbitrarily as a dichotomous marker for response since we found no published criteria for response on WSAS for PwP. In the only earlier PD treatment study using WSAS as an outcome, the adjusted mean change in WSAS from baseline corresponded to a 30% reduction in the treatment group, and a 3% increase in the waitlist group. [5] The possible difference in frequency of individuals who obtained a WSAS 30% reduction was tested for between groups with Fischer’s exact test. An exploratory *post hoc*-test examined if the frequencies of responders differed between the participants with a PD diagnosis for more than the overall median time (8 years), compared to those having received it more recently. This was to explore if outcome differed due to time since diagnosis, since a clinical impression during treatment was that more newly diagnosed participants tended to fare better in treatment.

## RESULTS

A majority of participants (61%) were women and the mean age was 66 years (age span 43–85 years). Average years since PD diagnosis were 8.9 (95% CI 7.8–10.0, median = 8). Average Levodopa Equivalent Dose of medication [33] were 999 mg (95% CI 865–1133, median = 928). Group comparisons of baseline characteristics are shown in Table 2. No significant differences between groups were found. Since all available time points were used in the GEE-analysis, the SCREEN time point was considered the baseline for WSAS and WHODAS-2 and the PRE time point for the remaining outcomes.

**Table 2:** Baseline characteristics of participants in ICBT and CONTROL groups

	ICBT (*n* = 38)	CONTROL (*n* = 39)
Women	24 (63%)	23 (59%)
Age at inclusion in years	65.9 (8.5)	66.1 (9.8)
Age span, youngest – oldest, in years	48–82	43–85
In a relationship	32 (84%)	25 (64%)
College/university educated	22 (58%)	20 (51%)
Working	4 (11%)	5 (13%)
Retired	26 (68%)	25 (64%)
Years since PD diagnosis	8.3 (4.4)	9.6 (5.7)
Levodopa equivalent dose	991 (544)	1006 (527)
Uses antidepressants	14 (37%)	10 (26%)

Data are number (%) or mean (SD). There were no significant differences between groups when testing with *T*-tests for continuous, and double-sided Fischer’s exact tests for categorical data.

### Primary outcome – daily functioning

There was a significant treatment effect on WSAS in the ICBT group compared to the control group, as shown by the significant interaction in Table 3, and the within-group change in WSAS score was also significant for the intervention group (W*χ*^2^= 17.38*, p* < 0.001). The calculated Bayes factor following a half-normal distribution was B H (0, 3.3) = 14.79 which is interpreted as evidence of a 3.3-point difference in WSAS over the null hypothesis of no difference.

**Table 3:** Estimated mean scores of primary and secondary outcomes, effect sizes and treatment group comparisons

	SCREEN m (SE)	PRE m (SE)	MID m (SE)	POST m (SE)	Effect sizes, *g* [CI] within-group	Effect size, *g* [CI] between-group	G*T *p*
Primary outcome
WSAS						0.69 [0.23, 1.15]	W*χ*^2^= 26.23
ICBT	25.71 (1.06)	22.92 (1.06)	22.13 (1.09)	21.48 (1.05)	0.64 [0.18, 1.10]		***p*** = **0.001**
CONTROL	25.84 (1.05)	24.84 (1.05)	26.04 (1.06)	26.04 (1.07)	–0.03 [–0.47, 0.40]
*p*	*p* = 0.934	*p* = 0.213	***p*** = **0.010**	***p*** = **0.002**
Secondary outcomes
HADS-A	–		–			0.51 [0.06, 0.96]	W*χ*^2^= 12.19
ICBT		7.79 (0.59)		6.87 (0.61)	0.25 [–0.21, 0.70]		***p*** = **0.007**
CONTROL		7.59 (0.58)		8.79 (0.59)	–0.33 [–0.77, 0.12]
HADS-D	–		–			0.68 [0.22, 1.14]	W*χ*^2^= 11.20
ICBT		7.34 (0.54)		6.36 (0.53)	0.29 [–0.16, 0.75]		***p*** = **0.011**
CONTROL		8.08 (0.53)		8.62 (0.53)	–0.16 [–0.61, 0.28]
ISI	–		–			0.38 [–0.07, 0.83]	W*χ*^2^= 18.92
ICBT		13.95 (0.95)		10.87 (0.97)	0.52 [0.06, 0.75]		***p*** = **<0.001**
CONTROL		13.51 (0.93)		13.15 (0.94)	0.06 [–0.38, 0.50]
PDQ-8	–		–			0.65 [0.19, 1.11]	W*χ*^2^= 15.55
ICBT		54.14 (1.79)		49.10 (1.83)	0.48 [–0.01, 0.90]		***p*** = **0.001**
CONTROL		54.87 (1.77)		56.45 (1.78)	–0.14 [–0.59, 0.30]
WHODAS-2			–			0.71 [0.25, 1.17]	W*χ*^2^= 17.22
ICBT	21.16 (1.07)	20.24 (1.07)		19.21 (1.09)	0.29 [–0.16, 0.74]		***p*** = **0.004**
CONTROL	22.10 (1.06)	22.23 (1.06)		23.97 (1.06)	–0.28 [–0.73, 0.17]
BBQ^a^	–		–			0.73 [0.26, 1.19]	W*χ*^2^= 13.03
ICBT		50.00 (3.04)		56.18 (3.15)	0.32 [–0.13, 0.77]		***p*** = **0.005**
CONTROL		41.77 (3.00)		42.12 (3.03)	0.02 [–0.43, 0.46]
SSES6^a^	–		–			0.23 [–0.21, 0.68]	W*χ*^2^= 1.29
ICBT		26.11 (1.72)		26.31 (1.81)	0.02 [–0.43, 0.47]		*p* = 0.732
CONTROL		24.97 (1.69)		23.72 (1.71)	–0.12 [–0.56, 0.33]

Bold values are statistically significant *p* < 0.05; ^a^Reversed scale (higher is better); WSAS, Work and Social Adjustment Scale; ICBT, treatment group; CONTROL, waitlist group; HADS-A, Hospital Anxiety and Depression Scale-Anxiety; HADS-D, Hospital Anxiety and Depression Scale-Depression; ISI, Insomnia Severity Index; PDQ-8, Parkinson’s Disease Questionnaire-8; WHODAS-2, World Health Organization Disability Assessment Schedule 2 - 12-item; BBQ, Brunnsviken Brief Quality of life scale; SSES6, Stanford Self-Efficacy for Managing Chronic Disease.

One third of the participants in the ICBT-group (12/37, 32%) were responders, compared to a tenth of participants in the control group (4/39, 10%). This was a statistically significant difference when tested with a double-sided Fischer’s exact test (*p* = 0.024).

The *post hoc* test, comparing participants having received their PD diagnoses for more or less than the median time of eight years, showed that those with a more recent diagnoses also had significantly better effect (*p* = 0.036) with more responders in the ICBT group (9/20; 45%) than in CONTROL (2/17; 12%). No significant difference in number of responders was found for participants receiving diagnosis 8 years ago or more (ICBT 3/17; 18% and CONTROL 2/21; 10%; *p* = 0.640). Around a quarter of the participants in the ICBT-group deteriorated, preliminary defined as having a higher WSAS score post treatment compared to baseline (10/37, 27%), compared to slightly over half of the patients on waitlist (22/39, 56%). This difference was statistically significant (double-sided Fischer’s exact test, *p* = 0.012).

### Secondary outcomes

For the secondary outcomes, significant differences were noted between groups (Table 3) for anxiety (HADS-A), depression (HADS-D), insomnia severity (ISI), Parkinson-specific functioning (PDQ-8), general disability (WHODAS-2), quality of life (BBQ), but not in self-efficacy in relation to chronic disease (SSES6). When examining the within-group effects on secondary outcomes in the ICBT-group, there was significant change only in HADS-D (W*χ*^2^= 4.70, *p* = 0.030), ISI (W*χ*^2^= 12.80, *p* = <0.001), PDQ-8 (W*χ*^2^= 7.91, *p* = 0.004) and BBQ (W*χ*^2^= 4.40, *p* = 0.036), but not in HADS-A (W*χ*^2^= 3.75, *p* = 0.053), WHODAS-2 (W*χ*^2^= 5.58, *p* = 0.061) or SSES6 (W*χ*^2^= 0.01, *p* = 0.915).

Clinician-rated status and change in the treatment group were as following: Clinical global impression CGI-S, m (SD) [95% CI] = 3.52 (0.93) [3.18–3.86], Clinical global impression CGI-I 2.97 (1.11) [2.56–3.37]. Eleven participants rated (35.5%, 11/31) were considered as much, or very much, improved. In addition, two participants rated with CGI-I (6.5%, 2/31) were rated as being worse off after the intervention. Eight participants (23.5%, 8/34) in the intervention group reported at least one adverse event at post. Many adverse events reported were due to filling out questionnaires, but there were also examples of adverse events relating to the exercises of the intervention. Thirty participants (88.2%, 30/34) reported at least one positive event during the intervention at post. Please see Table 4 for examples of the adverse and positive events reported.

**Table 4:** Three representative quotes from participants in the intervention group, post treatment relating to evaluation, adverse and positive events. Same row does not indicate that the quote is from the same participant

Subjective evaluation of the intervention	Reported adverse events	Reported positive events
“I have learned a lot about myself and how I react.”	“Sometimes I feel stress when answering the questionnaires. It takes more time than you think. Especially when you start and then get motor fluctuations, or the phone rings, someone comes to visit and so on.”	“I have, for example, taken the initiative to contact friends whom I neglected. This happened during the first three weeks of treatment.”
“Time was a little too short for each module.”	“The first part of the post-survey, which contained deeply personal questions, gave me nightly worries and I regretted that I answered.”	“I have sometimes initiated difficult conversations with my husband, without, like before, wait for his initiative.”
“If I could have been physically active, I would probably have seen more positive effects from the treatment. I know how important it is to be active and it is frustrating when you can’t.”	“There was a temporary undesirable effect of the meditation exercises. [...] It had given me acute bad stomach and discouragement for a few hours.”	“Documenting the number of steps per day has been very positive. It inspires me to walking up the stairs and not taking the elevator, both at work and in my home.”

### Feasibility in intervention group

Patient involvement and satisfaction (ICBT-group) was as following: modules completed, M (SD) [95% CI] = 7.55 (3.22) [6.49–8.61], sent messages 25.53 (13.33) [21.14–29.91], received messages 31.34 (8.66) [28.50–34.19], and treatment satisfaction with CSQ-8 26.25 (4.52) [24.62–27.88]. The proportion who completed at least the first four modules was 89%. Many quotes from the subjective evaluation of the intervention were positive regarding the intervention itself, but reported frustration and disappointment when the participant experienced failure to adhere to the homework of the intervention. Examples of these quotes can be found in Table 4. The mean self-rated benefit of using the activity meter to increase physical activity in the intervention group was (m [95% CI]): 2.62 [2.17–3.06] with 56% answering 3 or 4, meaning that they experienced much, or very much, benefit of using the activity meter. A quarter of participants (24 %) interviewed by phone post treatment declared that they had received some kind of other non-pharmacological intervention during the treatment period, but this was predominately physiotherapy and in no cases psychological treatment.

### Delayed treatment of waitlist, with support on demand

Estimated marginal means for WSAS during the delayed treatment of the control group were (M (SE)): 26.05 (1.02) before treatment, 25.37 (1.06) mid treatment and 25.18 (1.03) after delayed treatment. This did not correspond to a significant within-group change on WSAS (W*χ*^2^= 1.39, *p* = 0.499). The only secondary measure that showed a significant within-group change was insomnia severity (Estimated marginal means (SE)): 13.39 (0.93) before treatment and 11.60 (1.01) after treatment, W*χ*^2^= 4.70, *p* = 0.030).

Patient involvement and satisfaction (control-group) was as following (m (SD) [95% CI] *t*-test vs intervention group): modules completed = 5.26 (3.53) [4.11–6.40] *t* = 2.98 *p* = 0.004, sent messages = 10.69 (6.24) [8.67–12.72] *t* = 6.28 *p* < 0.001, received messages = 15.97 (5.81) [14.09–17.86] *t* = 9.17 *p* < 0.001, and treatment satisfaction CSQ-8 = 22.80 (5.20) [20.66–24.94] *t* = 2.67 *p* = 0.010. The proportion who completed at least the first four modules was 79%. The mean self-rated benefit of using the activity meter to increase physical activity in the control-group after delayed treatment was (m [95% CI]): 1.84 [1.34–2.34] with 39% answering 3 or 4, meaning that they experienced much, or very much, benefit of using the activity meter.

## DISCUSSION

This study investigated the effects of ICBT aimed at improving daily functioning for PwP, in adjunct to standard medical treatment. The results suggest that ICBT was more effective than standard medical treatment alone, with moderate between-group effect sizes for the main outcome and most secondary outcomes. Approximately one third of participants receiving the intervention had at least a 30% reduction of self-reported problems with general function. A similar proportion of participants in the intervention group were also considered as much, or very much, improved by the clinician, although the clinician-rated improvement ratings can be considered problematic because of lack of blinding and only being done in the treatment group. One third being considered as improved after ICBT is arguably somewhat low, compared to almost 60% considered improved in an earlier trial of CBT for depression in PD [4], but can be compared to results from CBT for epilepsy where a similar proportion of around a third was considered improved. [34] The effect sizes of *g* = 0.69 for functioning and *g* = 0.68 for depressive symptoms were similar to the preliminary effects on the main outcome HADS seen in the feasibility study (HADS *g* = 0.75, HADS-A *g* = 0.38, HADS-D *g* = 1.02), but considerably lower than the effect for depressive symptoms (*d* = 1.59) seen for face-to-face CBT for depression in PD by Dobkin and colleagues [4]. In WSAS, a marked reduction between screening and pre-treatment in the ICBT-group only (see Table 3) makes the results more difficult to interpret. Since the reduction happened before randomization it raises the question of the difference between groups of the main outcome WSAS being a chance finding. The differences between groups were on the other hand not significant at screening and pre-treatment, but became significant first at mid- and post-treatment (Table 3).

An exploratory *post hoc*-analysis suggested that it was participants having had PD for the shortest time that improved the most (45% versus 18%). This can be interpreted as the intervention possibly being more suited for PwP at an earlier stage. Average years since PD diagnosis was 8.9 years (95% CI 7.8–10.0) compared to 8.1 in the earlier feasibility study and 6.3 in the study by Dobkin and colleagues [4]. Levodopa Equivalent Dose was 999 mg (95% CI 865–1133) compared to 835 mg in the earlier feasibility study [13], suggesting a somewhat more severe level of PD in the current study. This raises questions on a possible mismatch between the intervention and the current sample included in the study that had quite severe levels of PD.

With regards to the secondary outcomes, there were significant within-group improvements as well as between-group effects in depressive symptoms, Parkinson-specific functioning, insomnia severity and general quality of life. For anxiety symptoms and general disability however, the significant effects are not accompanied with a significant improvement in the ICBT-group, suggesting the effect could be partly explained by deterioration in the control group as discussed above. The somewhat weak results on anxiety symptoms differs from those seen in a small preliminary study of CBT (face to face and videoconferencing) for people with anxiety and PD where the results on anxiety were promising [35]. Another small study of telephone-delivered CBT for anxiety and depression in PD, did however conclude that the intervention reduced symptoms of depression, but not anxiety [9]. Effects on depressive symptoms but not anxiety was also seen in our earlier feasibility study [13], altogether giving the impression that anxiety in PD might need extra consideration. Also, although HADS has been considered a useful anxiety measure, there may be difficulties with HADS in a PD setting, such as some items being insensitive to change because of their wording [36].

Patient involvement and treatment satisfaction in the ICBT-group were higher than in the feasibility study [13], and also compared to ICBT with on demand-support only given to participants initially on wait-list. The current satisfaction would be considered “excellent” according to Smith and colleagues [28], compared to only “moderate” for our feasibility study and “good” for the delayed ICBT with on-demand support. This, together with the lack of a significant increase in daily functioning after the wait-list received ICBT, suggest either the advantage of active therapist-support over on-demand support, or a negative effect of delayed treatment, or both. Deterioration in waitlist control conditions is a possibility in internet interventions [37] and highlights the need for a future study using an active control condition.

Participants’ subjective evaluations, and descriptions of adverse as well as positive events during treatment, illustrate that some participants benefitted from the interventions focus on behavioral activation and physical activity. There are, however, reports of frustration with aspects of the intervention, such as answering all the questionnaires and not being physically able to adhere to some advice on physical exercise. It is possible that further tailoring of the intervention to those with more severe cognitive or physical difficulties would ease some of this frustration.

Further randomized studies are needed to strengthen the support for this type of intervention, especially the *post-hoc* finding that it may be better suited for PwP with more recent PD-diagnosis. Developing an intervention which adapts to different levels of disease-severity could be highly relevant. The intervention could probably be further optimized to consist of the right package of efficient components and type of support, possibly using factorial experiment [38]. Another future opportunity is to further assess the benefit of caregiver support and to utilize caregivers more systematically in treatment.

### Strengths and limitations

This study is one of the largest randomized controlled trials of psychological treatment for people with PD and to our knowledge the largest one studying an internet-based intervention specifically adapted for this group of patients. A major strength was the very low attrition rate in the primary outcome measure (1%) and also the high involvement in treatment by the participants (89% completed 4 or more modules).

One limitation of the study was that the results might not be generalizable to everyone with PD as the participants were self-referred and only those with quite severe problems with daily functioning were included. Since the sample had low levels of employment, the WSAS could be less sensitive to treatment-related change than in other samples. Also, although participants with apparent difficulties using the technology associated with treatment were excluded from treatment, there was no screening for impaired cognitive function. The recruitment procedure demands logging in and filling out a lengthy screening questionnaire, and this to some extent automatically excludes individuals with severe cognitive difficulties, but participants with significant cognitive difficulties could still have been included in treatment and these problems could have contributed to non-response and frustration with treatment. However, feeling frustrated or stressed when trying to follow through with an ICBT program is common also in other patient groups [39]. There is also a possibility that the participants in the sample were an unusually highly motivated group since they in many cases were recruited from the Swedish Parkinson association. This could somewhat counter the speculated cognitive difficulties in the sample. Further, the sample consisted of a majority of women, which is a contrast to PD in general where men is a majority [40]. A further limitation is that there was no long time follow-up. Future studies of a revised ICBT-program may include this. Also, the additional comparison between active support and on-demand support is very preliminary since they were not compared directly. This limitation could be better addressed with a factorial experiment study design. Additionally, the use of a wait list in the control condition is sometimes associated with negative effects on symptoms. It is however important to note that in the context of this study, the control condition included publicly funded standard medical treatment. Further, there were no monetary incentives to any of the treatment groups, which would be a potential source of bias.

### Conclusions

In this study, a newly developed ICBT-program for people with PD and problems with daily functioning was found to be a feasible treatment alternative with moderate effects on functioning. The intervention may possibly be of larger benefit to people with more recently diagnosed PD than to those with longer since diagnosis. However, replications are needed and the treatment would probably benefit from further development and optimization to be able to help a larger proportion of PwP.

## CONFLICT OF INTEREST

The authors have no conflict of interest to report.
